# The association between gambling marketing and unplanned gambling spend: Synthesised findings from two online cross-sectional surveys

**DOI:** 10.1016/j.addbeh.2022.107440

**Published:** 2022-12

**Authors:** Heather Wardle, Nathan Critchlow, Ashley Brown, Craig Donnachie, Alexey Kolesnikov, Kate Hunt

**Affiliations:** a School of Social and Political Sciences, University of Glasgow, Adam Smith Building, Bute Gardens, Glasgow G12 8RT, United Kingdom; b Faculty of Public Health and Policy, London School of Hygiene and Tropical Medicine, 15-17 Tavistock Place, London WC1H 9SH, United Kingdom; c Institute of Social Marketing and Health, University of Stirling, Stirling FK9 4LA, United Kingdom

**Keywords:** Gambling, Advertising, Marketing, Emerging adults, Sports bettors, Surveys, Quantitative analysis

## Abstract

**Background:**

In 2020, the British Government initiated a review about whether to introduce stricter controls on gambling marketing. We examine: (i) what proportion of regular sports bettors and emergent adult gamblers report that marketing has prompted unplanned spend; and (ii) what factors are associated with reporting that marketing had prompted unplanned spend.

**Methods:**

Data are from two British non-probability online surveys with: (i) emerging adults (16–24 years; *n* = 3,549; July/August 2019) and (ii) regular sports bettors (18+; *n* = 3,195; November 2020). Among current gamblers, logistic regressions examined whether reporting that gambling marketing had prompted unplanned spend (vs never) was associated with past-month marketing awareness, past-month receipt of direct marketing (e.g., e-mails), following gambling brands on social media, and problem gambling classification.

**Results:**

Almost a third of current gamblers reported that marketing had prompted unplanned gambling spend (sports bettors: 31.2 %; emerging adults: 29.5 %). Escalated severity of problem gambling was associated with reporting that marketing had prompted unplanned spend in both samples, in particular those experiencing gambling problems compared to those experiencing no problems (sports bettors: *OR*_Adj_ = 17.01, 95 % CI: 10.61–27.27; emerging adults: *OR*_Adj_ = 11.67, 95 % CI: 6.43–21.12). Receipt of least one form of direct marketing in the past month and following a gambling brand on at least one social media platform was also associated unplanned spend among sports bettors and emerging adults.

**Conclusion:**

Among emerging adults and regular sports bettors, increased severity of gambling problems, receiving direct marketing, and following gambling brands on social media are associated with reporting that marketing has prompted unplanned spend.

## Highlights

• Marketing prompts unplanned spend in around a third of sports bettors and emerging adults.
• Most of those with gambling problems say marketing has prompted unplanned spend.
• Receiving direct marketing is also associated with reporting unplanned spend.
• Limiting exposure to gambling marketing may be a positive harm-reduction measure.

## 1 Introduction

There is growing international evidence that exposure to gambling marketing is a driver of gambling-related attitudes and behaviour, including links to likelihood of gambling, intentions to gamble, and gambling expenditure (Binde and Romild, 2019, Newall et al., 2019, Syvertsen et al., 2021, Rodda, 2020). In Britain, gambling marketing is well-resourced, with products promoted through a range of activities, including mass media advertising, sponsorship and endorsement, price offers and promotions, and digital marketing (Ginnis and Kitson, 2019, Torrance et al., 2021, Rossi et al., 2021). Data show these marketing activities are successful in reaching and engaging British consumers (Ginnis and Kitson, 2020, Torrance et al., 2021, Djohari et al., 2019). Consequently, the British Government, as elsewhere, are reviewing whether stricter controls on gambling marketing are necessary (Department for Digital, Culture, Media Sport. Policy paper: review of the Gambling Act, 2005). To inform this debate, we examine: (i) what proportion of regular sports bettors and emergent adult gamblers (aged 16–24 years) report that marketing has prompted unplanned spend on gambling and (ii) what factors are associated with reporting that marketing had prompted unplanned spend.

## 2 Materials and methods

### 2.1 Design and sample

Data come from two British surveys. The first is the Emerging Adult’s Gambling Survey, a longitudinal survey of 16–24-year-olds recruited in July/August 2019 (hereafter ‘emerging adults survey’) (Wardle, 2020). Analysis reported here uses wave one data (*n* = 3,549) to avoid the confounding influence of the COVID-19 pandemic. The second is a longitudinal survey of regular adult sport bettors (18+ years; who bet on sports at least monthly) (hereafter ‘sports bettors survey’) collected as part of ‘The Betting and Gambling COVID-19 impact study’ (Hunt et al., 2020). This analysis uses wave two data (*n* = 3,195; 82.6 % retention), collected in November 2020, as wave one data were collected during the early stages of the pandemic (July 2020) when restrictions on social movement may have impacted on both opportunities to gamble and exposure to marketing (Wardle et al., 2021). Wave two asked about experiences between August-October 2020 when all land-based gambling venues were open and live sports had returned. Copies of the sports bettor and emerging adults survey are available elsewhere (OSF, 2022a, OSF, 2022b).

For both surveys, the cohorts were recruited by YouGov from their non-probabilistic online panel of over one million members living in Britain. Participants were contacted by YouGov through direct e-mail invitations and received YouGov points (redeemable for vouchers) in remuneration. For both, a cross-sectional survey weight was provided to match the population profile of Great Britain with respect to age, sex, and region. For sports bettors, weights also matched the betting profile of regular gamblers. As there were only minor differences in survey design, we were able to compare between the two. The analyses focus on ‘current gamblers’, that is those undertaking any form of gambling in the last three months for regular sports bettors (*n* = 2,980; 93.2 % of wave two sample) or the last year for emerging adults (*n* = 1,496; 42.1 % of wave one sample).

### 2.2 Measures

#### 2.2.1 Unplanned spend on gambling being prompted by marketing activities

Sports bettors were asked ‘*Thinking about your gambling in the last three months, that is from August to October 2020, how often, if at all, did a gambling advert, promotion or sponsorship prompt you to spend money on gambling when you were not otherwise planning to?’* Similar wording was used in the emerging adults survey, except the timeframe was *‘in the last 12 months’*. Responses were provided on a four-point scale (1=’Very often’ to 4=’Never’). Responses were dichotomised into those who reported that marketing had prompted any unplanned spend (Very often/Often/Occasionally) versus those who did not (Never). The sports bettors survey also included a ‘Not sure’ option, which is excluded from analysis (*n* = 114).

#### 2.2.2 Past month awareness of gambling marketing

Awareness of gambling marketing was assessed using prompted recall, a frequently used method for examining consumer experiences of marketing exposure (Harris et al., 2006, Critchlow & Moodie, 2021) including gambling (Ginnis and Kitson, 2020). Participants in both surveys were presented with similar lists of marketing activities and asked *‘In the last month, have you seen or heard gambling being promoted in the following ways…’*. Participants ticked all that applied or ‘None of the above’. The emerging adults survey included eight activities and the sports bettors survey included ten. Both included adverts on television, radio, and social media; sport/event sponsorship; celebrity endorsement; online pop-up adverts, and adverts/promotions from gambling apps. The cumulative number of activities seen in the last month was calculated. For parity across surveys, total scores were grouped into low (0–1 activities), medium (2–3 activities) and high (≥4 activities) awareness, with each category representing around a third of responses in each sample (Table 1).

**Table 1:** Sample characteristics of current gamblers among regular sports bettor (Panel A) and emerging adults (Panel B).

	**Panel A: Regular sports bettors**					**Panel B: Emerging adults**			
	**Unweighted**		**Weighted**			**Unweighted**		**Weighted**	
**Variable**	***%***	***n***	***%***	***n***		***%***	***n***	***%***	***n***
**Sex**									
Male	80.9	2412	78.3	2299		48.8	730	54.9	829
Female	19.1	568	21.8	639		51.2	766	45.1	681
**Frequency of marketing prompting unplanned spend**									
Never	72.3	2073	68.8	1921		70.7	1058	70.5	1064
Occasionally	21.4	613	23.3	651		17.9	267	18.0	271
Often (Fairly often/Very often)	6.3	180	7.9	222		11.4	171	11.6	175
Not sure^1^	–	114	–	143		–	–	–	–
**Problem gambling (PGSI) category**									
Non-problem	68.5	2040	65.6	1927		59.5	890	58.7	886
Low risk gambler	17.5	520	17.2	506		23.5	352	24.2	366
Moderate gambler	8.6	258	10.2	301		8.2	123	8.3	125
Problem gambler	5.4	162	6.9	204		8.8	131	8.8	133
**Past-month gambling marketing awareness**									
Low (0–1 activities)	33.5	998	34.1	1001		33.2	497	32.8	495
Medium (2–3 activities)	30.2	900	30.4	892		29.1	436	28.3	427
High (≥=4 activities)	36.3	1082	35.6	1045		37.6	563	38.9	588
**Past month receipt of direct gambling marketing**									
None received	17.1	509	18.4	540		68.1	1018	67.6	1021
One marketing activity	16.4	489	15.7	461		22.6	338	22.5	340
Two or more marketing activities	66.5	1982	65.9	1937		9.4	140	9.8	148
**Follow gambling brands on social media**									
No	83.7	2494	78.5	2306		82.4	1233	81.4	1230
Yes – On at least one platform	16.3	486	21.5	631		17.6	263	18.6	280

**Notes:** Base = Regular sports bettors (A) = All those who had gambled in the past three months; Emerging adults (B) = All those who had gambled in the past 12 months; ^1^ ‘Not sure’ option was only included in the regular sports bettors survey and not emerging adults.

#### 2.2.3 Past month receipt of direct marketing from gambling companies

Receipt of direct marketing was also assessed using prompted recall. Participants were presented with a list of marketing activities and asked *‘In the past month, which of the following (if any) have any gambling companies sent directly to you?’* Participants ticked all that applied or ‘None of the above’. Both surveys included options for e-mails, text messages, social media messages, and notifications from a gambling app. Sports bettors were also asked about postal flyers/leaflets. Responses were summed to assess the cumulative number of direct marketing activities received in the past month. For parity across surveys, and to fit the differing distribution of responses among sports bettors and emerging adults (see Table 1), scores were grouped into participants who had received no direct marketing, one instance of direct marketing, or two or more instances.

#### 2.2.4 Following gambling companies on social media

Sports bettors were asked ‘*Do you follow or ‘like’ any gambling companies on any social media website or forum?’* Emerging adults were asked *‘Do you follow/watch gambling companies on any social media website or forum?’.* Both surveys included the clarification ‘*This includes companies who provide lottery games, bingo, betting, casino and slot games’.* In both, participants were presented with a list of social media platforms and asked to tick all that applied (e.g., ‘Yes, on Twitter’) or either ‘none’ or that they ‘do not use social media’. A binary variable was created indicating whether participants reported following/liking a gambling company on at least one platform (Yes/No).

#### 2.2.5 Problem gambling

In both surveys, participants completed the nine-item Problem Gambling Severity Index (PGSI) (Ferris and Wynne, 2001). Sports bettors were asked to think about the last three months. This timeframe intended to capture experiences since the first survey wave, approximately three months prior. A three month timeframe was also used in the first wave to capture data relating to the first COVID-19 lockdown in the UK (Wardle et al., 2021), which lasted approximately three months. While a shorter timeframe may be less sensitive to detecting gambling harms, versus reporting over a longer period, previous research has shown the utility of a shorter PGSI timeframe when assessing the impact of interventions (Abbott et al., 2012, Kushnir et al., 2018). Emerging adults were asked to think about the past 12 months. Items were scored on four-point scale (0=’Never’ to 3=’Almost always’), with a composite score (range 0 to 27) computed across items (Cronbach’s Alpha: regular sports bettors α = 0.948; emerging adults α = 0.937). Participants were grouped into non-problem gambling (0), low risk (1–2), moderate risk (3–7), and problem gambling (≥8).

### 2.3 Ethics

The sports bettors survey was approved by the University of Stirling’s General University Ethics Panel [GUEP:19/20–934]. The emerging adults survey was approved by the London School of Hygiene & Tropical Medicine’s Ethics Review Panel (REF:16023).

### 2.4 Analysis

Weighted frequencies examined the sample characteristics of current gamblers and the proportion who reported that marketing had prompted unplanned gambling spend. For both surveys, binary logistic regressions examined what factors were associated with reporting any unplanned gambling spend being prompted by marketing (‘any’ vs ‘never’). Covariates included PGSI category, awareness of gambling marketing (coded: low/medium/high), receipt of direct marketing (coded: none/one/two or more) and following any gambling companies on at least one social media platform (coded: Yes/No). Age, sex, educational status, employment status, and area deprivation were included as controls in both models. The data shown are the final stage main effects models. All analyses were performed using the complex survey function in Stata v15 to adjust for the weighted survey design.

## 3 Results

### 3.1 Associations with marketing prompting unplanned gambling spend: Sports bettors

In the regular sports bettors survey, 31.2 % of current gamblers reported that marketing had prompted unplanned gambling spend in the past three months (Table 1). This rose to 87.0 % among those experiencing problem gambling. After controlling for demographics, socio-economic and marketing exposure variables, PGSI status was associated with reporting that marketing had prompted unplanned gambling spend (Table 2; Panel A). Specifically, those experiencing problem (*OR*_Adj_ = 17.01, 95 % CI: 10.61–27.27), moderate risk (*OR*_Adj_ = 3.41, 95 % CI: 2.35–4.94), and low-risk gambling (*OR*_Adj_ = 3.31, 95 % CI: 2.58–4.26) were more likely to report that marketing had prompted unplanned spend than those experiencing no gambling problems.

**Table 2:** Associations between reporting that marketing had prompted unplanned gambling spend and problem gambling (PGSI) category, past-month marketing awareness, and engagement with marketing among both regular sports bettors (Panel A) and emerging adults (Panel B).

	**Panel A: Regular sports bettors^1,2^**				**Panel B: Emerging adults^3,4^**		
**Covariates**	***OR_Adj_***	***95 % CI***	***p***		***OR_Adj_***	***95 % CI***	*p*
**PGSI category**							
Non-problem	REF	–			REF	–	
Low risk gambler	3.31	2.58–4.26	<0.001		1.82	1.30–2.54	<0.001
Moderate gambler	3.41	2.35–4.94	<0.001		2.34	1.43–3.81	<0.001
Problem gambler	17.01	10.61–27.27	<0.001		11.67	6.43–21.12	<0.001
**Past-month marketing awareness**							
Low (0–1 activities)	REF	–			REF	–	
Medium (2–3 activities)	1.15	0.86–1.54	0.343		1.13	0.79–1.63	0.505
High (≤4 activities)	1.19	0.88–1.61	0.254		0.87	0.60–1.25	0.457
**Past-month receipt of direct marketing**							
None	REF	–			REF	–	
One form of direct marketing	3.20	2.39–4.30	<0.001		2.70	1.94–3.76	<0.001
Two or more instances of direct marketing	5.54	4.05–7.57	<0.001		3.55	2.15–5.85	<0.001
**Follow gambling brand on social media**							
No	REF	–			REF	–	
Yes – On at least one platform	1.45	1.08–1.90	0.015		3.15	2.17–4.59	<0.001

**Notes:** Dependent variable in both models = Self-reporting that marketing had prompted unplanned spend on gambling (Very often/Fairly often/Occasionally = 1) versus Never (=0); Main effects models, which control for sex, age, educational attainment, employment/educational status; and area level of deprivation (not reported here); Analyses are weighted; *OR_Adj_* = Adjusted Odds Ratio; 95 % CI = 95 % Confidence Interval for *OR_Adj_*; ^1^ Base: Regular sports bettors who had gambled in the past three months; ^2^ Data missing for sports bettor model (*n* = 114, ‘not sure’ on gambling prompting unplanned spend); ^3^ Base = Emerging adults who had gambled in the past 12 months; ^4^ Data missing for emerging adults model (*n* = 0).

Receipt of direct marketing in the past month and following or liking a gambling brand on social media were also associated with reporting that marketing had prompted unplanned spend (Table 2; Panel A). After controlling for demographics, socio-economic status and PGSI status, regular sports bettors who had received one form of direct marketing in the past-month (*OR*_Adj_ = 3.20, 95 % CI: 2.39–4.30), and those who had received two or more (*OR*_Adj_ = 5.54, 95 % CI: 4.05–7.57), were more likely to report that marketing had prompted unplanned spend than those who received no direct marketing. Similarly, regular sports bettors who said they followed or liked a gambling brand on at least one social media platform were more likely to report that marketing had prompted unplanned spend than those who did not follow or like on any platform (*OR*_Adj_ = 1.45, 95 % CI: 1.08–1.90). After controlling for participatory marketing variables (i.e., receipt of direct marketing/following brands on social media), past-month awareness of marketing was not associated with unplanned gambling spend among regular sports bettors.

### 3.2 Associations with marketing prompting unplanned gambling spend: Emerging adults

In the emerging adults survey, 29.5 % of current gamblers reported that marketing had prompted unplanned gambling spend in the past 12 months (Table 1). This rose to 87.0 % among those experiencing problem gambling. After controlling for demographic. socio-economic and other marketing variables, PGSI status was associated with reporting marketing had prompted unplanned gambling spend (Table 2; Panel B). Specifically, those experiencing problem (*OR*_Adj_ = 11.67, 95 % CI: 6.43–21.12), moderate risk (*OR*_Adj_ = 2.34, 95 % CI: 1.43–3.81), and low-risk gambling (*OR*_Adj_ = 1.82, 95 % CI: 1.30–2.54) were more likely to report that marketing had prompted unplanned gambling spend than those experiencing no gambling problems.

Receipt of direct marketing in the past month and following or liking a gambling brand on social media was also associated with reporting that marketing had prompted unplanned spend (Table 2; Panel B). After controlling for demographics, socio-economic status and PGSI status, emerging adults who had received one form of direct marketing in the past month (*OR*_Adj_ = 2.70, 95 % CI: 1.94–3.76), and those who had received two or more (*OR*_Adj_ = 3.55, 95 % CI: 2.15–5.85), were more likely to report that marketing had prompted unplanned spend compared to those who received no direct marketing. Moreover, emerging adults who said they followed or liked a gambling brand on at least one social media platform were more likely to report that marketing had prompted unplanned spend compared with those who did not like or follow on any platform (*OR*_Adj_ = 3.15, 95 % CI: 2.17–4.59). After controlling for participatory marketing variables, past-month awareness of gambling marketing was not associated with unplanned gambling spend among emerging adults.

## 4 Discussion

Among independent studies of emerging adults and regular sports bettors, almost a third of current gamblers, and almost nine-in-ten of those experiencing gambling problems, reported that marketing had prompted unplanned gambling spend. Among both samples, escalating problem gambling status, past-month receipt of direct marketing, and following or liking a gambling brand on at least one social media platform was associated with reporting that marketing had prompted unplanned spend.

Our analyses are cross-sectional and unable to demonstrate causality in the associations between experiencing gambling problems and reporting that marketing had prompted unplanned gambling spend. Nevertheless, the consistent presence and size of such associations in two independent samples suggests that marketing likely plays some role in problem gambling, and there are harm-reduction implications regardless of whether this is an initiating role (i.e., marketing prompting unplanned spend is a contributory factor to escalated gambling problems) or a reinforcing role (i.e., those already experiencing gambling problems are more susceptible to being prompted into unplanned spend by marketing). It is plausible this association is bidirectional, with the role varying among gambling subgroups. While longitudinal research should further investigate the direction of these associations, and possible mediating or moderating factors, the presence of any association between problem gambling and reporting that marketing had prompted unplanned spend suggests that restrictions on where, and how often, current gamblers are exposed to marketing may be a positive harm-reduction measure.

There are other limitations. From a sampling perspective, both studies use non-probability samples, which have attendant issues for generalisability. However, regular gamblers can be a hard-to-reach group and online panels have wider sample coverage for emerging adults than probability methods (Wardle, 2020). While non-probability panels are not advised for prevalence estimates, they perform better when focusing on the relationship between variables, as this study does (Callegaro et al., 2014). Sports bettors data were collected during the COVID-19 pandemic and, although Britain was not in full national ‘lockdown’ (Brown and Kirk-Wade, 2021), requirements for social distancing may have impacted on exposure to marketing and gambling behaviours (e.g., more online versus land-based). The emerging adults data were collected pre-pandemic.

Concerning measurement limitations, all estimates are self-reported and are susceptible to recall errors for marketing awareness, receipt of direct marketing, and the perceived influence of marketing on prompting unplanned spend. We also only captured data on any past-month awareness of marketing or any past-month receipt of direct marketing, but not frequency or volume. This reduces specificity in the associations between marketing exposure and reporting unplanned spend. Use of aggregate scores for past-month marketing awareness and receipt of direct marketing also means the data do not account for the impact of individual marketing activities. We also only measured whether any marketing had prompted unplanned spend, but did not collect data about what marketing features facilitated this, for example the presence of offers and inducements, advert design, or brand salience.

In conclusion, in two independent studies, around a third of current gamblers and the almost nine-in-ten of those experiencing problem gambling reported that marketing had prompted unplanned spend on gambling. In both sports bettors and emergent adults, such reports are associated with receiving more direct gambling marketing in the past month and following or liking a gambling brand on at least one social media platform. Longitudinal assessments of causality, and possible mediating or moderating factors, remain important areas for future investigation. A precautionary interpretation of these data, particularly the associations between problem gambling and marketing prompting unplanned spend, suggests that restrictions on gambling marketing may be a positive harm-reduction measure.

## Funding Source

The sports bettors survey was funded by the Economic and Social Research Council, ref no: ES/V004549/1. The emerging adults survey was funded by Wellcome Trust, ref no: 200306.

## CRediT authorship contribution statement

**Heather Wardle:** Conceptualization, Data curation, Formal analysis, Funding acquisition, Investigation, Methodology, Project administration, Supervision, Validation, Writing – review & editing. **Nathan Critchlow:** Conceptualization, Funding acquisition, Methodology, Project administration, Supervision, Writing – original draft, Writing – review & editing. **Ashley Brown:** Methodology, Project administration, Writing – review & editing. **Craig Donnachie:** Data curation, Methodology, Project administration, Validation, Writing – review & editing. **Alexey Kolesnikov:** Formal analysis, Writing – review & editing. **Kate Hunt:** Funding acquisition, Methodology, Project administration, Supervision.

## Declaration of Competing Interest

HW was Deputy Chair of the Advisory Board for Safer Gambling between May 2015 and March 2020. She was remunerated by the Gambling Commission (the industry regulator) for this. She is a member of the WHO panel on gambling and in 2018/19 worked on a study looking at gambling and suicide funded by GambleAware. NC has worked on one study funded by GambleAware, which examined the impact of gambling marketing/advertising on young people and vulnerable groups. CD, AB, AK and KH have no competing interests to declare.
